# Localisation of immune cell populations during human fetal testicular development

**DOI:** 10.1530/RAF-25-0189

**Published:** 2026-08-05

**Authors:** Samira Hosseini, Davor Jezek, Marina Kos, Benedict Nathaniel, Ewa Rajpert-De Meyts, Mark P Hedger, Hans-Christian Schuppe, Kate L Loveland, Daniela Fietz

**Affiliations:** ^1^ Centre for Endocrinology and Reproductive Health, Hudson Institute of Medical Research, Clayton, Victoria, Australia; ^2^ Department of Molecular and Translational Sciences, Monash University, Clayton, Victoria, Australia; ^3^ Department of Histology and Embryology, Centre of Excellence for Reproductive and Regenerative Medicine, School of Medicine, University of Zagreb, Zagreb, Croatia; ^4^ Department of Pathology, School of Medicine, University of Zagreb, Zagreb, Croatia; ^5^ Department of Growth and Reproduction, Copenhagen University Hospital – Rigshospitalet, Copenhagen, Denmark; ^6^ Department of Urology, Pediatric Urology and Andrology, Justus Liebig University Giessen, Giessen, Germany; ^7^ Institute of Veterinary Anatomy, Histology and Embryology, Justus Liebig University Giessen, Giessen, Germany

**Keywords:** immune cells, testis, human fetal development

## Abstract

**Graphical Abstract:**

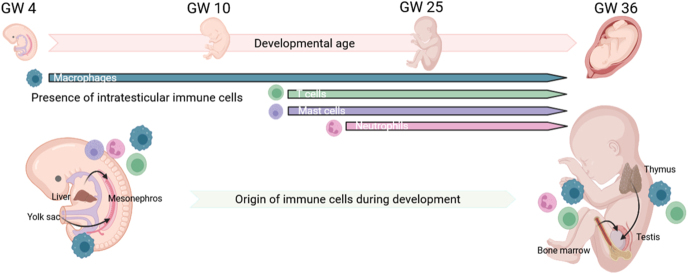

**Abstract:**

This study addresses the limited knowledge of immune cells and their origins in the human fetal testis. By identifying and localising macrophages, T cells, neutrophils, and mast cells, evidence of their potential roles in testicular and epididymal development is provided. Macrophages (CD68+), T cells (CD3+), neutrophils (CD66b+), and mast cells (tryptase+ and chymase+) were identified using immunohistochemistry and immunofluorescence in 20 formalin-fixed fetal testes (and epididymides, when present) collected at autopsy from stillborn fetuses (gestational weeks, GW14–41). Immune cell transcripts were compiled from published scRNA-seq datasets from human embryonic (GW6–8) and fetal (GW12–16) testes. CD68+ macrophages were abundant in interstitial, testis border, and perivascular regions, increasing adjacent to cords from GW23 onward. Interstitial CD3+ T cells were rare in second trimester but more frequent in the third. CD66b+ neutrophils were extremely rare. Chymase+ mast cells were absent, while tryptase+ mast cells were present near the capsule and vessels. scRNA-seq identified CD68-expressing cells as the predominant immune population from GW6–16. This first description of multiple immune cell types in the human fetal testis reveals similarities to murine testes; in both species, early macrophage abundance, preceding bone marrow haematopoiesis, suggests yolk sac and/or liver origins, while later-appearing immune cells are likely bone marrow-derived. The near-complete absence of Ly6G+ neutrophils in the later gestation stages differs from observations in mouse. These findings highlight potential developmental windows vulnerable to immune-related disruption and reinforce the value of mouse models for understanding human testis development.

**Lay summary:**

This study examines how the immune system helps the testicles to form before birth. In animals, immune cells called macrophages are important for early testis development, but their roles and those of other immune cells in humans were unclear. We studied tissues from 20 stillborn babies between 14 and 41 weeks of pregnancy to identify immune cells in developing testes and compared our results with existing genetic data. We found that macrophages were the most common immune cells, appearing early and increasing in number as the testis grew. Other immune cells, including T cells and mast cells, appeared later. Our findings suggest that macrophages come from early sources, such as the yolk sac or fetal liver, and play a key role in building the testis. Understanding these processes could reveal how factors affecting immune cell functions before birth could impair male fertility in men.

## Introduction

Macrophages are the most abundant immune cells in adult testes of humans and rodents, and studies of their distribution and function revealed their vital contributions to male fertility. In adult mice, two distinct macrophage populations exhibit different functional markers (DeFalco *et al.* 2015, Mossadegh-Keller *et al.* 2017, Mossadegh-Keller & Sieweke 2018, Indumathy *et al.* 2020). Interstitial macrophages reside between seminiferous tubules close to Leydig cells, while peritubular macrophages surround the tubules alongside peritubular myoid cells (Bhushan *et al.* 2020). These macrophages perform two crucial functions: supporting the immune-privileged microenvironment in the presence of germ cell ‘neo-antigens’ and delivering pro-inflammatory signals in response to infection (Bhushan & Meinhardt 2017, Bhushan *et al.* 2020). In addition, macrophages help maintain testosterone production by Leydig cells (Hedger 2015) and influence spermatogonial stem cell behaviour (DeFalco *et al.* 2015, Heinrich & DeFalco 2020). Other immune cells are normally present at a much lower frequency, including dendritic cells (DCs), mast cells and/or eosinophils, T cells, and natural killer (NK) cells, with B cells rarely observed (Hedger 2015, Klein *et al.* 2016, Bhushan *et al.* 2020, Islam *et al.* 2024). Amongst these, T cells, neutrophils, and mast cells contribute to immune privilege maintenance and are involved in the immune responses affecting function in the human adult testes (Fijak & Meinhardt 2006, Klein *et al.* 2016, Yamada *et al.* 2016, Himelreich-Perić *et al.* 2022, Islam *et al.* 2024).

Knowledge of immune cell distribution and function during testis development employing mouse studies is relatively new. We now understand the central importance of different immune cells, particularly macrophages, to fetal testicular cord formation, testosterone production, immune privilege, and maintenance of ongoing spermatogenesis into adulthood (Fijak & Meinhardt 2006, DeFalco *et al.* 2014, 2015, Lokka *et al.* 2020, Gu *et al.* 2022, 2023, Hosseini *et al.* 2022). For both ethical and practical reasons, little is known about the human fetal testis where prolonged gestation could challenge mother and fetus and alter systemic and local functions. This study provides a basis for considering the key roles of immune cells in earliest stages of human testis formation that lay the foundation for adult fertility and hormone production.

Primordial germ cell migration into the bipotential gonad from the yolk sac wall via the mesonephros is followed closely by the events of sex determination, which occurs in humans at gestational weeks (GW) 4–6. Testicular cords start to form around GW6–7, as Sertoli cells proliferate and establish the first developmental niche for fetal gonocytes or pre-spermatogonia by enveloping them into cords. After sex determination, immune cells initially represent a small fraction of the total cell number, but their number and proportional representation gradually increase. By GW14–18, the gonocytes begin the transition into a quiescent state, forming spermatogonial precursors that underpin lifelong spermatogenesis, while the somatic cell population continues to expand (Li *et al.* 2017, Guo *et al.* 2021). In the mouse fetal testis, macrophages are required for normal cord formation (DeFalco *et al.* 2014).

Functionally distinct macrophage populations emerge progressively during fetal life preceding full development of the adaptive immune system, and these are originally either yolk sac- or fetal liver-derived (DeFalco *et al.* 2014, Lokka *et al.* 2020, Wang *et al.* 2021, Meinhardt *et al.* 2022). The majority of macrophages in the adult testis originate from monocytes that ultimately form both interstitial and peritubular populations (Mossadegh-Keller *et al.* 2017, Gu *et al.* 2023). These tissue-resident macrophages are long-lived and capable of self-renewal (Bhushan & Meinhardt 2017). While the role of macrophages in organ development has been deduced in the mouse, it remains to be established in humans (DeFalco *et al.* 2014, Miah *et al.* 2021). In human fetal testes, macrophages have recently been identified from single-cell transcript profiling of first- and early-second-trimester gonads (Garcia-Alonso *et al.* 2022). However, there is almost no information about the emergence and localisation of macrophages and other immune cell subtypes in human fetal testes, despite their likely importance in the key steps of testis growth that determine adult fertility. Amongst other immune cells, studies of neutrophils in fetal testes are limited to two investigations in the mouse (DeFalco *et al.* 2014, Hosseini *et al.* 2022). Datasets generated through scRNA-seq (e.g. Park *et al.* 2020, Guo *et al.* 2021, Garcia-Alonso *et al.* 2022) have provided tools with which we can deduce the developmental trajectory of human immune cell population emergence in the context of organ development. However, to understand their particular functional relevance during periods of dynamic growth, it is critical to document their distribution. In the present study, we assessed the frequency and localisation of immune cells in the human fetal testis using immunohistochemistry (IHC) on archival human fetal testis samples from second to third trimesters and compared this with scRNA-seq data (Guo *et al.* 2021). This approach provides an assessment of the emergence of key immune cell types, yielding new information about their population size and positions to indicate how immune cells may contribute to the earliest stages of human testis development.

## Materials and methods

### Samples

Human fetal testes were collected during routine paedopathological autopsy of spontaneously aborted/stillborn fetuses between GW14 and GW41 at the Department Histology and Embryology, School of Medicine, University of Zagreb, Croatia, and the Departments of Growth and Reproduction and Pathology, Rigshospitalet, University of Copenhagen, Denmark. The age of archive samples fixed in 4% paraformaldehyde (PFA, w/v) was in the following ranges: GW14/15–20 (*n* = 8), GW21–24 (*n* = 8), GW25 (*n* = 3), GW33–34 (*n* = 3), and GW41 (*n* = 1). All investigations were performed after obtaining written informed consent according to the Declaration of Helsinki. All procedures were approved by the local ethics committees in the Department Histology and Embryology, University of Zagreb, the Capital Region of Denmark, and the University of Copenhagen.

### Immunohistochemistry

IHC was performed using antibodies to detect all immune cells (anti-CD45, protein encoded by gene *PTPRC*), macrophages (anti-CD68), T cells (anti-CD3), mast cells (anti-tryptase, gene *TPSAB1*; anti-chymase, gene *CMA1*), and neutrophils (anti-CD66b, gene *CEACAM8*) (antibodies listed in Table 1). Sections were cut, deparaffinised, rehydrated, and placed in deionised water for 3 min. Sizes of sections used can be taken from Supplementary Table 1 (see section on Supplementary materials given at the end of the article). Heat-induced antigen retrieval in citrate buffer (pH 6.0) (Sigma-Aldrich, USA) was followed by incubation with 3% (v/v) hydrogen peroxide (Merck Millipore, USA) for 25 min at room temperature (RT), one wash in Tris-buffered saline (TBS; 50 mM Tris-Cl, 150 mM NaCl, pH 7.5) containing 0.1% (v/v) Triton X-100 for 3 min, and then two washes for 3 min with TBS. Sections were next incubated in blocking solution (5% bovine serum albumin, BSA; Sigma-Aldrich) in TBS at RT for 1 h. Primary antibodies diluted in 1.5% BSA/0.1% Triton X-100/TBS (w/v) were applied overnight at 4°C. Omission of primary antibody was used for negative controls. Slides were washed and then incubated with secondary antibodies for 1 h at RT. After washing, Vectastain Elite ABC kit (Vector Laboratories, USA) was applied for 30 min at RT. Antibody binding was identified using DAB (Agilent Technologies, USA). After counterstaining with haematoxylin (Sigma-Aldrich), slides were washed and dehydrated through an increasing ethanol gradient and histosol. Sections were mounted using DPX (Sigma-Aldrich).

**Table 1:** Primary and secondary antibodies used for IHC and IF.

	Company	Cat No.	Clone	Raised in	Dilution
Primary antibodies					
Anti-CD45	DAKO, USA	M0701	2B11+ PD7/26	Mouse	1:250
Anti-CD3	DAKO, USA	0452	Polyclonal	Mouse	1:150
Anti-CD68	DAKO, USA	M0876	PG-M1	Mouse	1:250
Anti-tryptase	DAKO, USA	7,052	AA1	Mouse	1:200
Anti-tryptase	Abcam, UK	Ab2378	AA1	Mouse	1:8,000
Anti-chymase	Abcam, UK	Ab233103	Polyclonal	Mouse	1:200
Anti-CD66b	BioLegend, USA	G10F5	G10F5	Mouse	1:200
Anti-laminin	Sigma-Aldrich, USA	L9393	Polyclonal	Rabbit	1:250
Secondary antibodies					
Rabbit anti-mouse biotinylated	DAKO, USA	E0468	Polyclonal	Rabbit	1:500
Goat-anti mouse HRP-conjugated	Dianova, Germany	115-035-003	Polyclonal	Goat	1:400
Donkey anti-Rabbit Alexa Fluor 488	Invitrogen, USA	A21202	Polyclonal	Donkey	1:500
Donkey anti-Mouse Alexa Fluor 555	Invitrogen, USA	A31572	Polyclonal	Donkey	1:500

### Immunofluorescence

Indirect immunofluorescence (IF) was performed using antibodies to detect all immune cells, macrophages, and the cord boundary (anti-laminin), as previously described (Whiley *et al.* 2023). Antibodies are listed in Table 1. Deparaffinisation, dehydration, and antigen retrieval were performed as for IHC. Slides were permeabilised using 0.1% Triton X-100/PBS for 5 min at RT and then washed in PBS, and blocking solution (5% donkey normal serum, in 10% BSA/PBS w/v) was applied for 1 h at RT. Primary antibodies were diluted in 5% BSA/0.1% Triton X-100/PBS, and slides were incubated overnight at 4°C. Negative control sections lacked primary antibody. Slides were then washed in 0.1% Triton X-100/PBS and PBS, and conjugated secondary antibodies were applied for 1 h at RT, with slides protected from light from here. Then, the slides were processed as above. DAPI (4′,6-diamidino-2-phenylindole; Invitrogen (USA), D3571, 1:500 (v/v) in PBS) was used to visualise chromatin/nuclei. Slides were mounted using ProLong Gold Mountant (Invitrogen) and stored at RT for 30 min and then at 4°C until image analysis.

### Imaging and morphometric analysis

Human fetal testis sections were scanned using an Olympus VS120 Slide Scanner at the MHTP Monash Histology Platform and analysed using OlyVIA Software (Olympus Life Science (Japan), 2.9.1 Viewer). Background staining and non-specific signals were excluded from counting using the negative controls. Immune cells were identified by size, nuclear morphology, and distinct markers (Supplementary Fig. S1). Immune cell localisation within each section was defined as ‘border’ (between the capsule and cords) or ‘inner area’ (containing the interstitium and cords). A more refined scheme further distinguished ‘interstitium’ (area between cords), ‘testis perimeter’ (outside the cords), ‘inside vessels’ (blood or lymphatic), ‘cord perimeter’ (close to the cord wall and peritubular cells), and ‘inside cord’.

### Cell type identification and clustering using Seurat

To analyse single-cell RNA sequencing (scRNA-seq) data from human fetal testes (Guo *et al.* 2021), the Seurat (Butler *et al.* 2018, Stuart *et al.* 2019) pipeline (http://satijalab.org/seurat/, R package, v.4.0) was employed. Data retrieved from the Gene Expression Omnibus (GEO) repository consisted of 10X Chromium datasets (Guo *et al.* 2021) for GW6–8, 12, 15, and 16 in market exchange (MEX) format (Accessions GSE143356). The raw unique molecular identifier (UMI) count tables were loaded into R using the Read10X function. Individual Seurat objects were created for each age: GW6–8 (‘embryonic’) and GW12, 15, and 16 (‘fetal’). These were filtered, merged, and log-normalised with the default settings as described in the original study. Next, cells were normalised to the total UMI read count and mitochondrial read percentage (http://satijalab.org/seurat/). Cell clustering and UMAP analyses were performed based on the statistically significant principal components. All software (R Studio, USA) and packages were publicly available from CRAN (Seurat). All graphs were generated by the Seurat package in R. Cell type analyses based on top marker expression were performed using EnrichR (Supplementary Table 4).

## Results

### Immunohistochemical and IF analysis of immune cells in fetal testis and epididymis

This study used human fetal testis sections (and epididymis, if present) spanning the second and third trimesters of pregnancy to identify the location and relative frequency of immune cell populations by IHC/IF. A summary of analysed sections and section sizes can be taken from Supplementary Table 1. A set of markers was used to provide a broad representation of key immune cell types, i.e. CD45^+^ pan-leucocytes, CD68^+^ macrophages, CD3^+^ T cells, tryptase and chymase^+^ mast cells, and CD66b^+^ neutrophils. A summary is provided in Table 2.

**Table 2:** Semi-quantitative analysis of immune cell numbers and distribution in human fetal testis sections.

Sample			Immune cell distribution				
Marker	Immune cell	Pregnancy trimester	Interstitium	Testis perimeter	Inside vessels	Cord perimeter	Inside cords
CD45^+^	Leucocytes	2nd	++	+	+	++	−
		3rd	+++	++	++	+++	−
CD68^+^	Macrophages	2nd	++	++	−/+	++	−
		3rd	+++	+++	−/+	+++	−
CD3^+^	T cells	2nd	−/+	−	+	−/+	−
		3rd	+/++	−	++	+	−/+
CD66b^+^	Neutrophils	2nd	−	−	+	−	−
		3rd	+	−	++	−/+	−
Tryptase^+^	Mast cells	2nd	−	+	−	−	−
		3rd	−	++	−	−	−
Chymase^+^	Mast cells	2nd	−	−	−	−	−
		3rd	−	−	−	−	−

Absent: −; rare (1–5 cells/section): +; frequent (5–20 cells/section): ++; abundant (>20 cells/section): +++. Section area average size and range: second trimester = 6,907,044.44 μm^2^ (range: 30,250.00–12,768,755.7); third trimester = 35,787,466.02 μm^2^ (range: 97,220.00–98,682,440.10).

**Figure 1:**
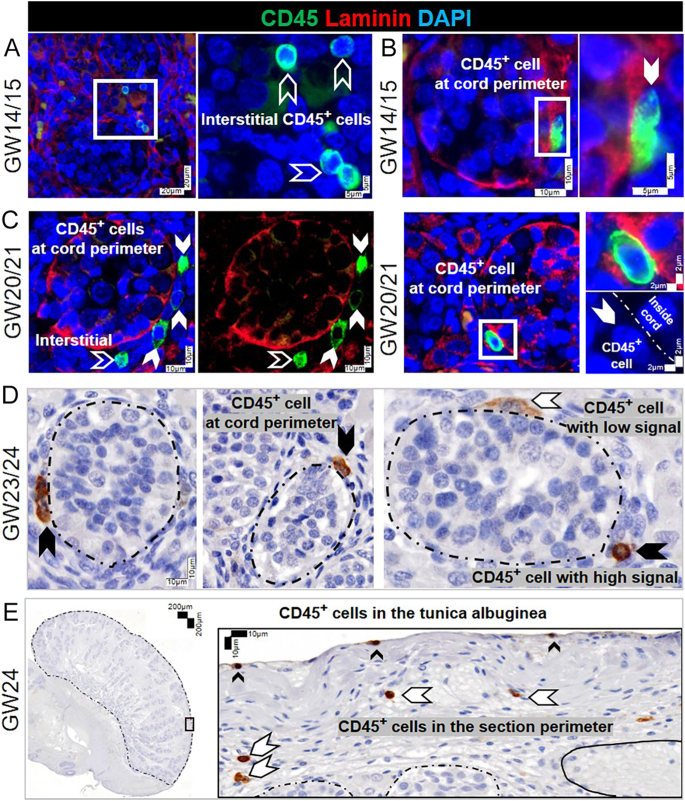
CD45^+^ cells in human fetal testis sections. CD45^+^ cells (green) were identified in the interstitium during GW14–15 (A, white-lined arrows) and cord perimeter at GW14–15 (B) and GW20–21 (C, filled white arrows). Nuclear staining with DAPI and tubular wall staining for laminin in red. (D) White arrows indicate a weak staining, while black arrows indicate a strong staining of CD45^+^ leucocytes in the cord perimeter at GW22–23. (E) CD45^+^ leucocytes were found in the tunica albuginea (black arrows) and in the section borders (white arrows). (D and E) Nuclear staining with haematoxylin. Black and white boxes on low-magnification images refer to adjacent high-magnification panels. Each panel represents an individual sample. Testis and cord perimeter are indicated with a dotted line, and blood vessels are indicated with a line.

Detection of CD45 yielded an initial overview of the distribution and abundance of immune cells in the human fetal testis. During the second and third trimesters, CD45^+^ cells were abundant in the testis interstitium, adjacent to the cord wall (Fig. 1A, B, C, D), and close to the tunica albuginea (Fig. 1E). CD45^+^ cells in the epididymis appeared more abundant during second and third trimesters compared to the testis, but were absent from areas close to epididymal ducts (Supplementary Fig. S2).

**Figure 2:**
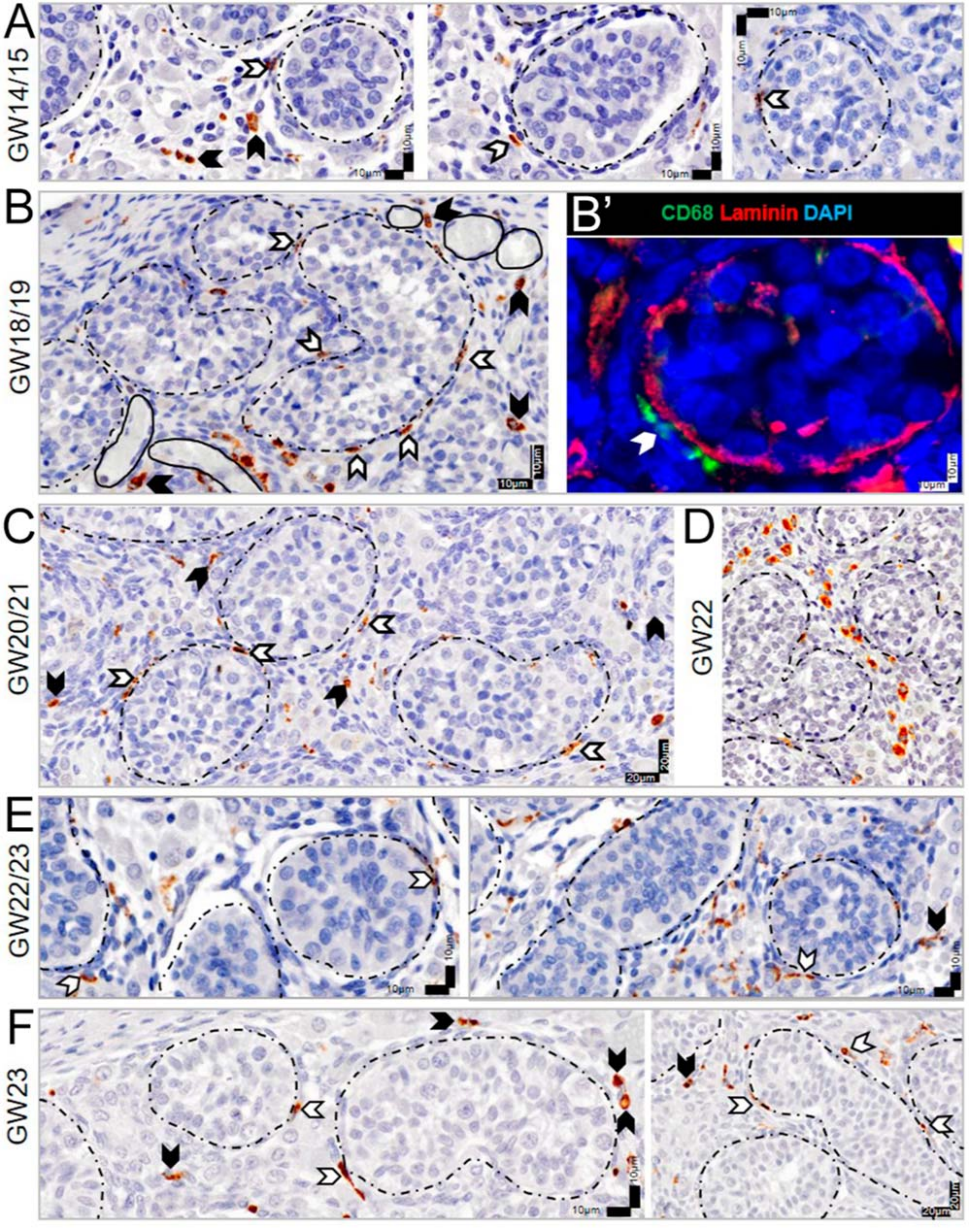
CD68^+^ macrophages are detected in cord perimeter (white arrow) and the interstitium (black arrow) of human fetal testis sections. CD68^+^ macrophages were detected at (A) GW14–15, (B) GW18–19, (C) GW20–21, (D) GW22, (E) GW22–23, and (F) GW23. Double IF staining of CD68 (macrophages, green) with laminin (tubular wall, red) was added for better orientation of fetal cord architecture (B). Note the orientation of interstitial macrophages along septa at GW22 (D). IHC: nuclear counterstain with haematoxylin; cord perimeters are indicated with a dotted line.

CD68^+^ macrophages were abundant in the interstitium and testicular border, prominent especially around vasculature, but extremely rare to absent inside blood vessels or inside cords (Fig. 2). Macrophages were present adjacent to cords from GW14–15. After GW22, macrophages were localised along the forming connective tissue septa (Fig. 2D). After GW23, macrophages were found in all locations (Fig. 2E). CD68^+^ cells were also frequent in the epididymis, but were not observed in close proximity to the ducts (Supplementary Fig. S3 and S4).

**Figure 3:**
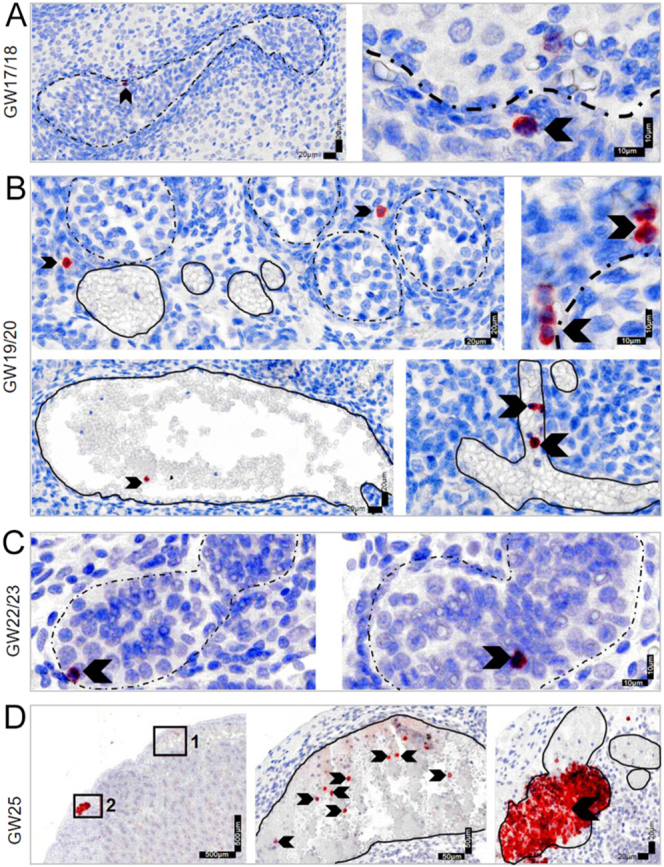
CD3^+^ T cells in human fetal testis. (A) At GW17–18, CD3^+^ cells were detected in cord perimeter. (B) At GW19–20, more CD3^+^ cells were detected in the interstitial compartment and in cord perimeter. Additionally, CD3^+^ cells were detected inside blood vessels. (C) At later stages, GW22–23, CD3^+^ cells were also detected inside the cords. (D) At GW25, CD3^+^ cells were detected in large numbers inside blood and lymph vessels, mainly at the border of the tissue section. CD3^+^ cells are indicated by black arrows. Dimensions as indicated. Cord perimeters are indicated with a dotted line, and blood vessels are indicated with a line.

CD3^+^ cells were rare in the second trimester but more frequent during the third trimester in the interstitium. The majority were located close to blood or inside lymph vessels (Fig. 3). In addition, CD3^+^ T cells were close to testicular cords in most samples but rarely observed inside the cords in individual samples during the second trimester of pregnancy (Fig. 3). A small number of CD3^+^ cells were detected in the tunica albuginea and rete testis.

**Figure 4:**
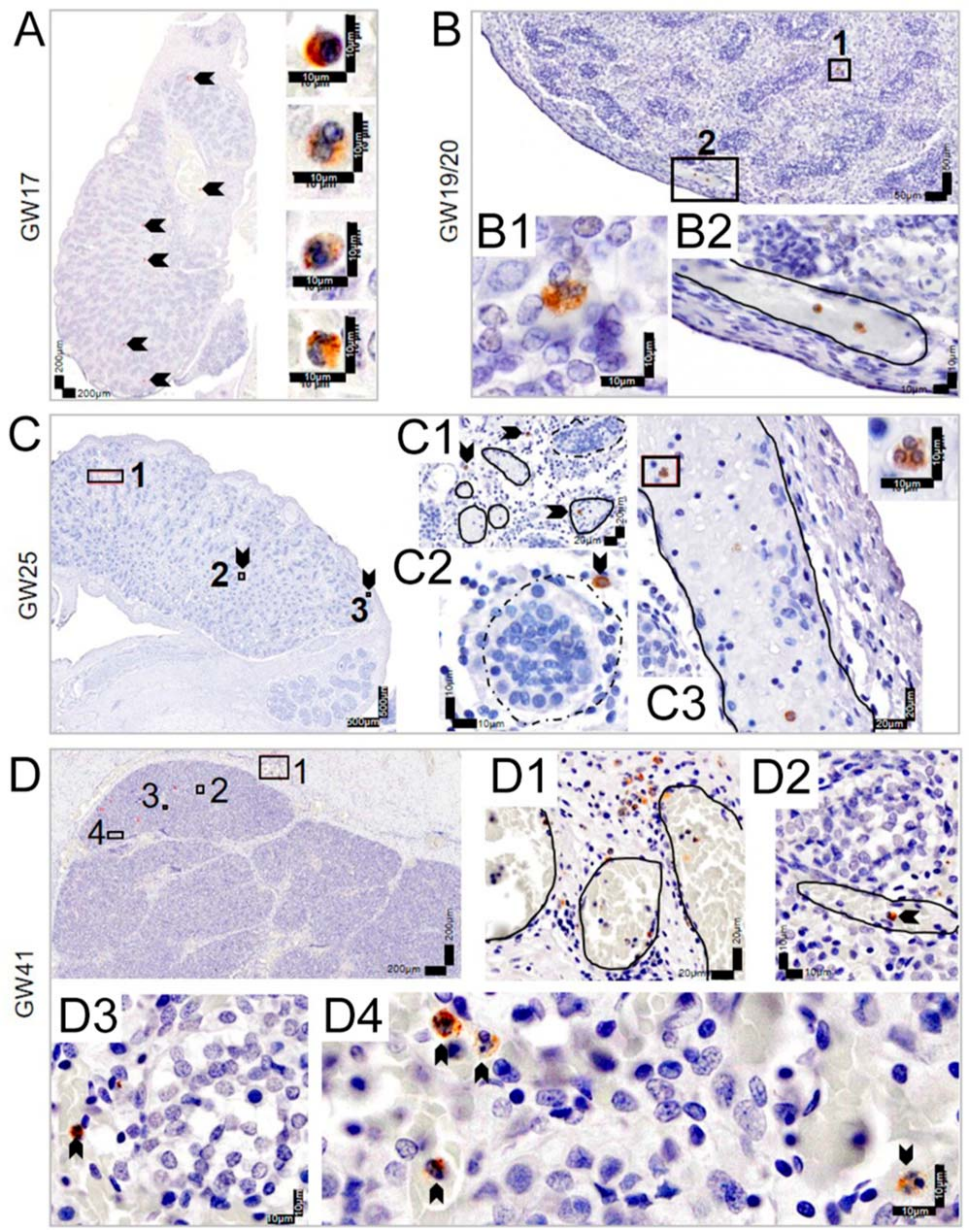
CD66b^+^ neutrophils in human fetal testis. C66b^+^ cells were generally rare. CD66b^+^ cells are indicated using black boxes and arrows at GW17–18 (A), at GW19–20 (B), and at GW25 (C). C66b^+^ neutrophils are shown in higher magnifications in panels A and C. (D) At GW41, CD66b^+^ cells were present in the testis section interior, most frequently around blood vessels, and in the epididymal side of the testis. Numbered black boxes in low-magnification images are shown in higher-magnification panels. Dimensions as indicated. Cord perimeters are indicated with a dotted line, and blood vessels are indicated with a line.

CD66b^+^ neutrophils were extremely rare in the testicular interstitium and absent from the epididymis from GW17 to GW25, with only individual CD66b^+^ cells identified inside blood vessels. At GW41, CD66b^+^ cells were numerous around vessels on the epididymal side of the testis and present in the testicular interstitium (Fig. 4). In the epididymis at this age, neutrophils were abundant in the caput region but absent from the corpus and cauda (Supplementary Fig. S5).

**Figure 5:**
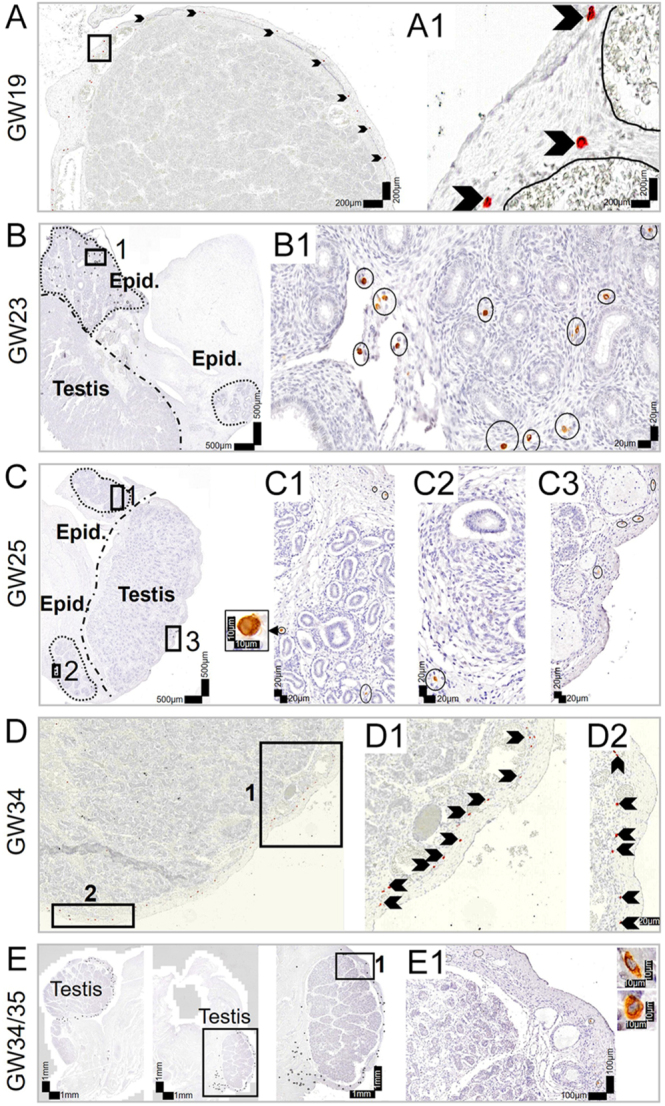
Tryptase^+^ mast cells in human fetal testis. (A) At GW19, tryptase^+^ mast cells were detected only in the testis perimeter, between the capsule and the outermost cords (arrowheads); higher magnification in A1. (B and C) At GW23–25, more tryptase^+^ mast cells were found in testis-adjacent epididymal tissue (Epid.) but remained close to the capsule in the testis; higher magnification in B1. (D and E) At GW34–35, mast cells were still evident around the testis perimeter. Black arrowheads or circles indicate tryptase^+^ mast cells. Numbered black boxes in the low-magnification images are shown in higher-magnification panels. Dimensions as indicated. Cord perimeters are indicated with a dotted line, and blood vessels are indicated with a line.

Chymase^+^ mast cells were not detected in the human fetal testis at any age examined. Tryptase^+^ mast cells were not identified inside the fetal testis or inside blood vessels during the second and third trimesters, but were present in testis border area close to the capsule, as well as around vessels. In contrast, they were frequently visible on the epididymis side of the testis and within the epididymis (Fig. 5).

### Single-cell RNA-sequencing data analyses

Examination of publicly available scRNA-seq data (Guo *et al.* 2021) allowed evaluation of immune cell transcript levels and distribution during the embryonic and fetal phases of testicular development. We collated data about transcripts selectively expressed in macrophages (*CD68, CD14, CD206 (MRC1), MHC class II (HLA-DRB1, HLA-DRA), CD163, CD64 (FcγRI), CD80, CD86 (B7-2), CD115 (CSF1R)*), monocytes (*CD14, CCR2 (MCP-1-R)*), T cells (*CD3E, CD3G, CD3D, CD4, CD8A*, *CD25 (IL2RA), FOXP3, CTLA-4 (aka CD152), CD28*), mast cells (*tryptase (TPSAB1), chymase (CMA1)*), neutrophils (*CD33, CD66b (CEACAM8), CD15 (FUT4), CD16 (FCGR3A), MPO*), B cells (*CD19, CD20 (MS4A1)*), DCs (*CD1C, CD141 (THBD), CD11C (ITGAX), CD303 (CLEC4C)*), and natural killer cells (*CD56 (NCAM1), CD94 (KLRD1), CD16 (FCGR3A), NKG2A/CD159a*). We extracted additional markers for pan-leucocyte (*CD45 (PTPRC)*), proliferation (*Ki-67*), and immune checkpoints (*PD-1* and *PD-L1)*. Lists of all embryonic and fetal markers and of top markers for both datasets are supplied in Supplementary Tables 2, 3, 4.

**Figure 6:**
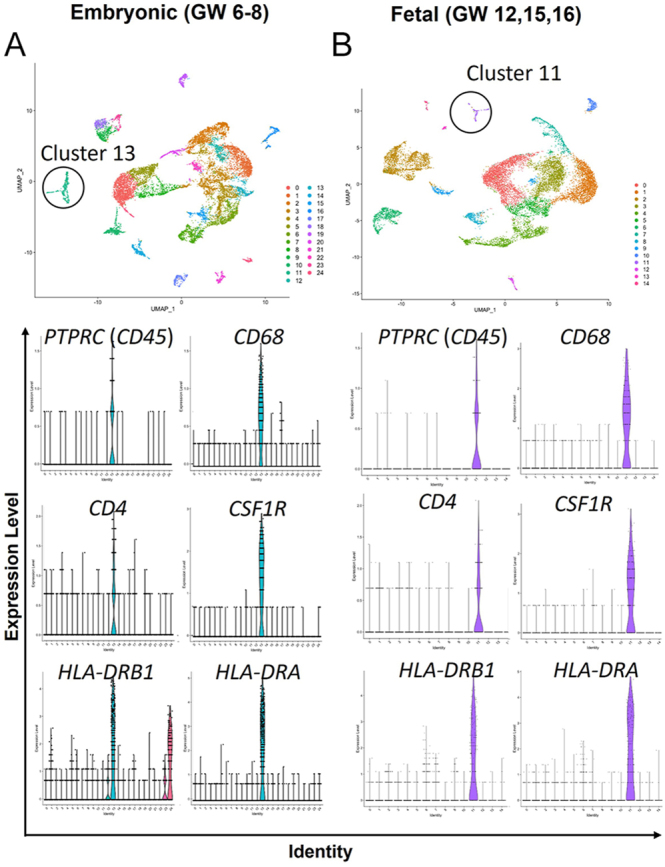
Transcript data from human embryonic (GW6–8, A) and fetal testis (GW12, 15, and 16, B) scRNA-seq (Guo *et al.* 2021). Cell clusters (UMAP) were generated by combining single-cell transcriptome data from embryonic (GW6–8, A) and fetal (GW12, 15, and 16, and B) samples, *n* = 1 for each age. Below UMAP, violin plots represent immune cell marker levels within individual cells in each cluster. Solely cluster 13 in the embryonic dataset and cluster 11 in the fetal dataset contained *PTPRC*, encoding *CD45* (pan-leucocytes). Expression of *CD68* (macrophages), *CD4* (macrophages and T helper cells), *CSF1R* (monocytes and macrophages), and *HLA-DRB1* and *HLA-DRA* (antigen-presenting cells) is shown for both embryonic and fetal samples. Colours indicate cluster identity (green: cluster 13 of embryonic samples; violet: cluster 11 of fetal samples). Individual points represent a single cell. Colour intensity scales indicate relative transcript levels. Violin plots illustrate immune cell marker levels within individual cells in each cluster (*n* = 1 for each age).

We identified a single cluster (cluster 13 in the embryonic dataset and cluster 11 in the fetal dataset) comprised of cells expressing *PTPRC* (*CD45*) (Fig. 6). These were verified as immune cells by the profiles of other markers, which were barely detected, or not detected, in other clusters (Supplementary Tables 2, 3, 4). In both age groups, the number of *CD68^+^* cells in cluster 13 (the embryonic testis dataset) or 11 (the fetal testis dataset) was much greater than other immune cell markers, as expected based on immunohistochemical detection of macrophages. Levels of *HLA-DRB1, HLA-DRA, *and* CSF1R* transcripts were higher than those of *CD206, CD163, CD64,* and *CD86*, while the *CD80* level was extremely low for embryonic samples and undetectable for fetal ones. The monocyte marker CCR2 was extremely low in both datasets (Fig. 6, Supplementary Fig. S6 and S7).

To examine T cells, we used *CD3*, *CD8*, and *CD4*. There were few cells encoding *CD3E, CD3D, CD3G, CD8A*, and *CD8B* in both age groups with significantly higher numbers of *CD4*^+^ cells (Fig. 6, Supplementary Fig. S6 and S7), most likely being macrophages also expressing *CD4* (Zhen *et al.* 2014). Only very low levels of the *CD28* transcript were present, while *FOXP3* and *CTLA-4* transcripts were also almost undetectable (Supplementary Fig. S6 and S7).

*TPSAB1* (encoding mast cell tryptase), *CD1C* (DC marker), and *CD20* were barely detected but present in cluster 13 or 11 (Fig. 6). Transcript markers for other cell types, such as NK (*NKG2A/CD159A*), neutrophils (*CD66B, CD15, CD16,* and *MPO*), immune checkpoints (*PD-1* and *PD-L1*), and proliferation (*MKI67*), were absent (Supplementary Fig. S6 and S7).

## Discussion

Little is currently known about the identity and function of testicular immune cells during human fetal morphogenesis. The increasing understanding of how disturbances in embryonic development can lead to defective germ cell development and subsequent sub- or in-fertility provides the motivation to establish the key events that are vulnerable to disruption. Building on the expanding knowledge of how immune cells contribute to fetal testis growth from studies in the mouse, this study presents the first systematic examination of key immune cell types that emerge during the second and third trimesters of human pregnancy. The integration of findings using immunohistochemistry and IF on tissue sections with published scRNA-seq data (Guo *et al.* 2021, Sararols *et al.* 2021) has provided a timeline for comparing the immune cell composition during mouse and human fetal testis development. For both histological and transcript samples, we analysed embryonic samples from GW6–8; fetal samples from GW12, 15, and 16; and samples from later gestational periods (second and third trimesters of pregnancy). This not only enabled the identification and spatial distribution of immune cells but also offered information about their origin. Whereas immune cells present at GW6–8 are yolk sac-derived, data generated from second and third trimesters also include bone marrow-derived immune cells (see Graphical Abstract). Because most tissue-resident immune cells in the steady state are derived from embryonic progenitors (Hoeffel *et al.* 2012, Kleer *et al.* 2014, Hoeffel & Ginhoux 2015, Lokka *et al.* 2020, Wang *et al.* 2021), a description of immune cells present during fetal testis development can shed light on the origin of resident immune cells in later life.

Analysis of the transcript encoding the common leucocyte marker CD45, *PTPRC* (Kaplan *et al.* 1990), provides a broad overview of immune cells. During embryonic/fetal development, *CD45^+^* cells were abundant in one specific cluster containing predominantly macrophage signatures. In the later stages of testicular development, which includes immune cells originating from the bone marrow, abundant CD45^+^ cells were detected by immunohistochemistry in the interstitium, in and around vessels, around and adjacent to cords, and in the rete testis.

Most CD45^+^ cells were identified as CD68^+^ macrophages with both approaches, making them the most prevalent immune cell population during embryonic and fetal development, whereas other immune cell types were much less frequent. During the second trimester, they were abundant in the testis perimeter, interstitium, and around vessels in the fetal testis, while being extremely rare or absent within blood vessels. From GW14–15 to GW41, their frequency adjacent to seminiferous cords and apparent number in the epididymis increased. The origin of macrophages is distinct in different tissues in mice, with microglia, Langerhans cells, alveolar macrophages, and Kupffer cells first originating from yolk sac progenitors, followed by the emergence of fetal liver-derived monocytes. Macrophages in the heart, pancreas, gut, and dermis initially originate from fetal liver monocytes but are substantially replaced by bone marrow-derived monocytes later in life (Wolf *et al.* 2019). This provides the basis for understanding the dynamic embryonic ontogeny of human testicular macrophages. The scRNA-seq data from both embryonic and fetal testes confirm the abundance of macrophage marker transcripts *CD68, CD14*, and *CSF1R* in cluster 13 or cluster 11 and indicate the prevalence of this cell type in the fetal testis (Guo *et al.* 2021). Isolation and scRNA-seq analysis of CD45+ cells from various human fetal organs, including the testis (6–12 PCWs), identified two macrophage populations distinguished by the presence of transcripts encoding either *SIGLEC15* or *TREM2*. During this interval of rapid testis growth, their differing positions within the interstitium or cords were demonstrated using single-molecule fluorescence *in situ* hybridisation (Garcia-Alonso *et al.* 2022). Functional engagement in tissue remodelling, cell migration, and direct or indirect communication with Sertoli and germ cells was proposed based on their individual transcript profiles. Additionally, scRNA-seq data also provide evidence of the potential functional status of fetal testis macrophages. Embryonic and fetal testis scRNA-seq data confirmed that a high number of cells expressed *HLA-DR* (*HLA-DRA and HL-DRB1*) and *CD14* transcripts. HLA-DR proteins are required for antigen presentation to T cells, permissive for either inducing an immune response or suppressing T cell activation (Bright & Munro 1981). Fetal liver-derived macrophages (*CD14^+^*) at 6–17 PCWs exhibit high *HLA-DR* levels on monocyte/macrophage progenitors (Popescu *et al.* 2019). Those expressing *HLA-DRA* and *HLA-DRB1* (predominantly found on M1 macrophages) were more abundant than those with *CD206* and *CD163* (predominant on M2 phenotype macrophages). In the mouse fetal testis, the MHC class II protein is not detected on testicular macrophages before birth (Mossadegh-Keller *et al.* 2017, Lokka *et al.* 2020, Hosseini *et al.* 2022). Although *HLA-DR* is also present in B cells and DCs, we are confident that its presence represents the detection of macrophage transcripts in these early testis ages, as the numbers of B cells and DCs were low during the second trimester. This provides a point of contrast between mouse and human fetal testes regarding immune cell composition. Additional investigations are needed to evaluate the phenotypic and functional similarities and differences between mice and humans regarding MHC class II (HLA-DR) expression on macrophages. HLA-DR proteins require CD80 and CD86 as co-stimulatory molecules to present antigens to T cells in order to regulate their activation status (Halliday *et al.* 2020). However, in human fetal testis samples, the number of cells expressing the *CD86* transcript was low, and *CD80* was undetectable in this study. This suggests that testis macrophages before GW16 may not be proficient at antigen presentation.

Detection of low T cell transcripts in embryonic and fetal testis agrees with previous studies. T lymphocyte progenitor colonisation of the human thymus occurs during GW8, and the first mature T cells are detected in the thymus at GW13 (Stites & Pavia 1979, Haynes *et al.* 1989). *CD3*^+^ cells are present in the human fetal liver from GW7–8, and those with *CD8* mRNA are detectable at 12–14 PCWs (Lobach & Haynes 1987, Haynes & Heinly 1995, Farley *et al.* 2013, Popescu *et al.* 2019). T cell (CD3^+^ and CD4^+^CD25^+^ cells) spreading from the thymus to the periphery starts rapidly after GW13, while CD3^+^ T cells are observed in peripheral blood not earlier than GW15–16. The presence of CD3^+^ cells in the thymus and periphery occurs concordant with the appearance of regulatory T cells (Tregs and CD4^+^CD25^+^ cells), indicating a tight control loop of fetal immune responses (Darrasse-Jèze *et al.* 2005). CD3^+^ cells and Tregs are detectable in the fetal spleen, liver, bone marrow, and intestinal mucosa between GW14.5 and GW16 (Spencer *et al.* 1986, Royo *et al.* 1987, Darrasse-Jèze *et al.* 2005).

From GW14 onward, Tregs in fetal organs are functional and express intracellular cytotoxic *CTLA-4* and *FOXP3* mRNA (Darrasse-Jèze *et al.* 2005). Fetal Tregs have been of interest, as an *in vitro* study demonstrated that human fetal CD4^+^ cells preferentially polarised into Tregs after stimulation against non-inherited maternal alloantigens. This immune suppression is critical during pregnancy, and interestingly, the regulated immune response against maternal alloantigens continues even during adulthood (Mold *et al.* 2008).

The current study demonstrates that CD3^+^ cells are rare compared to macrophages and mainly scattered in the interstitium, in the proximity of or inside blood vessels, and at low numbers around the cords during the second trimester, with an increase in number during the third trimester of pregnancy, with a more pronounced presence in the cord perimeter and occurring rarely inside seminiferous cords. While some CD45^+^ cells were identified with T cell markers in the fetal testis (Garcia-Alonso *et al.* 2022), their functional relevance remains to be established in any species for this organ.

Tissue-resident mast cells in adult human tissues are typically categorised as either mucosal or connective tissue mast cells; whereas the first exclusively produce tryptase, the second produce both tryptase and chymase (Krystel-Whittemore *et al.* 2015). Studies in mice have demonstrated that mucosal mast cells are T cell-dependent for activation, can induce inflammation, and exclusively originate from bone marrow-derived progenitors. In contrast, connective tissue mast cells seeded during fetal morphogenesis are exclusively derived from embryonic progenitors (mainly fetal liver) and are maintained at a steady state (Kitamura *et al.* 1977, Heng & Painter 2008, Dwyer *et al.* 2016, Li *et al.* 2018). In the human fetus, tryptase-expressing mast cells are present in the yolk sac at 4–7 PCWs and fetal liver at 7–8 PCWs (Popescu *et al.* 2019). Later, but before birth, they distribute, forming local, self-renewing mast cell progenitor pools that are sustained throughout adulthood. However, the predominant source of mast cell progenitors in adults is bone marrow (Popescu *et al.* 2019). In samples of the second and third trimesters, tryptase^+^ mast cells were abundant both below the tunica albuginea and in the epididymis. The low number of tryptase^+^ cells in the testis was confirmed by scRNA-seq data. Due to the age of testes and the presence of mast cells in mainly connective tissue-rich areas, we deduce that fetal testes (and epididymis) are populated by embryonically derived mast cells, with mucosal mast cells being recruited from the bone marrow later.

Neutrophilic myeloid cells are derived from yolk sac blood islands and fetal liver (Popescu *et al.* 2019), but most neutrophils emerge during the bone marrow phase of haematopoiesis during fetal development (Koenig & Yoder 2004). At 10–11 PCWs, neutrophils are detectable within the human clavicular marrow cavity for the first time (Charbord *et al.* 1996, Lawrence *et al.* 2018) with mature neutrophils identified in peripheral blood by 14–16 PCWs. This timeline explains why CD66b^+^ neutrophils were rare to absent in the fetal testis and epididymis before GW41 in our study, in agreement with scRNA-seq data. Other markers of neutrophil status, such as CD15, CD16, and MPO, were virtually absent in both datasets (Guo *et al.* 2021).

At 7 PCWs, immature DCs are detected in the fetal liver and non-lymphoid organs, such as skin and kidney (Popescu *et al.* 2019). Functional DCs are detectable in human skin and liver by GW13, mainly inducing immune suppression by interacting with activating Tregs. We searched for various DC markers in the scRNA-seq dataset, finding only few cells expressing *CD1C, CD141,* and *CD303* and some more showing *CD11C.* It would be interesting to identify DC protein markers in the second and third trimesters of pregnancy in future analyses.

B cells are first present in the human fetal liver at 8 PCWs. Their frequency is very low before 12 PCWs, but increases thereafter (Popescu *et al.* 2019). Our study and scRNA-seq data (Guo *et al.* 2021, Garcia-Alonso *et al.* 2022) identify only a low proportion of B cells in the human fetal testis.

NK cell precursors originate in the yolk sac and fetal liver; upon populating target organs, they differentiate *in situ* and acquire tissue-related gene expression profiles (Popescu *et al.* 2019). As NK cell markers were very rare or absent in the scRNA-seq datasets, we propose that only few NK cells reach the fetal testes earlier than GW16, although the application of immunohistochemistry should be considered for older testis samples.

Finally, we identified *PD-1* transcript-expressing cells in fetal, but not embryonic testes (Guo *et al.* 2021). PD-1 and its ligand, PD-L1, are expressed on activated immune cells (T cells, B cells, monocytes, and macrophages) and non-haematopoietic cells. PD-1 acts on T cells to induce Treg development, suppressing immune responses to chronic inflammation and inhibiting immune responses against autoantigens. During human fetal development, PD-1 in the context of the fetal–maternal interface appears important for induction of tolerance (Qin *et al.* 2019). Although the *PD-1* transcript is rarely detected on unstimulated primary T cells (Yamazaki *et al.* 2002), its presence in the human fetal testis is intriguing.

## Conclusion

This study combined protein and transcriptional data to analyse the ontogeny and localisation of immune cell populations in the human embryonic and fetal testes. As in mouse, macrophages are the most abundant immune cell type in human fetal testis with distinct phenotypes and patterns. Macrophages are the main cell type driving extracellular matrix remodelling during human fetal testis organogenesis. The localisation of CD45^+^ cells and macrophages in the cord perimeter areas suggests that these cells may contribute to germ cell differentiation and cord formation, as recently proposed in mouse and other human studies (Garcia-Alonso *et al.* 2022, Hosseini *et al.* 2022). Differences in macrophages and tryptase^+^ mast cell numbers in testis and epididymis indicate different immune physiology as demonstrated in adult tissues (Sullivan *et al.* 2019). This study provides a framework for further investigations into immune cell ontogeny and function in the male reproductive system and their importance for human fertility.

## Supplementary materials























## Declaration of interest

The authors declare that there is no conflict of interest that could be perceived as prejudicing the impartiality of the work reported.

## Funding

This work was supported by the Deutsche Forschungsgemeinschaft (DFG), Monash University (GRK 1871/2), on ‘Molecular pathogenesis on male reproductive disorders’, and Centre of Excellence for Reproductive and Regenerative Medicine (European Union Regional Development Fund, Operational Program Competitiveness and Cohesion, under grant agreement PK.1.1.10.0003, ‘Development of innovations and translational research in reproductive and regenerative medicine’). KL was supported by NHMRC grants (Fellowship: 1079646; Projects: 1081987 and 1181516). MH was supported by NHMRC grants (Fellowship: 1020269 and Project Grant: 1063843). KL and MH received support from the Victorian State Government Operational Infrastructure Scheme to the Hudson Institute of Medical Research.

## Author contribution statement

All authors contributed to the intellectual content of the manuscript and approved the final version. SH performed the experiments; BN performed the scRNA-seq data extraction; SH, BN, KL, DF, MH, and H-CS analysed the data; SH and BD prepared the figures; and SH and KL drafted the manuscript. DJ and ERD provided samples and scientific support. SH, KL, MH, DF, and HCS were responsible for the conception and design.
