# Supplementary material for: Localisation of immune cell populations during human fetal testicular development
Source: Reprod Fertil. 2026 Aug 5;7(3):RAF250189. doi: 10.1530/RAF-25-0189 (PMC13452043; doi:10.1530/RAF-25-0189)
Supplement: Supplementary file 1 [file RAF-25-0189_supplementary_figure_1.pdf]

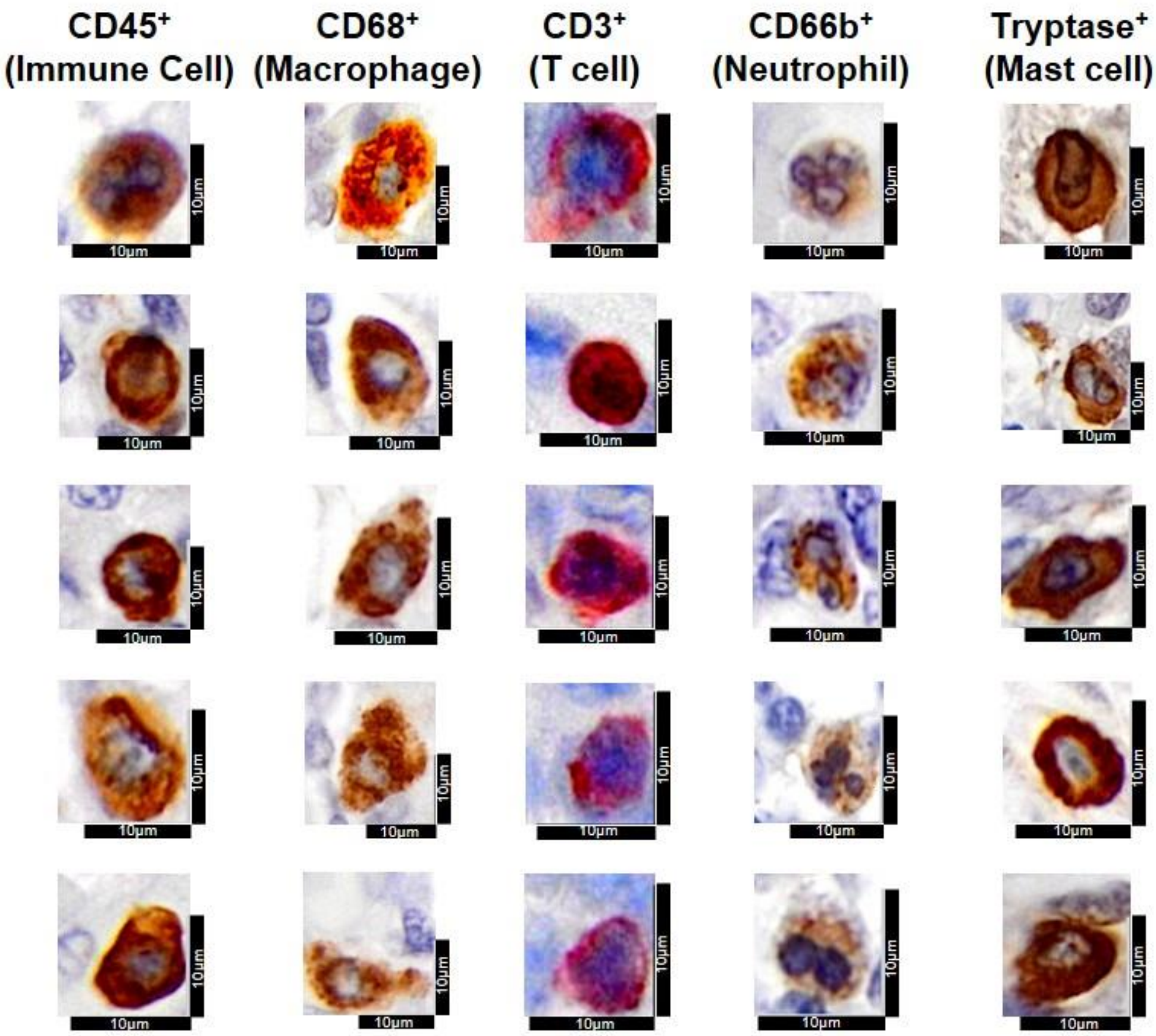
CD45+ (Immune Cell)
CD68+ (Macrophage)
CD3+ (T cell)
CD66b+ (Neutrophil)
Tryptase+ (Mast cell)
10µm
