# Supplementary material for: Localisation of immune cell populations during human fetal testicular development
Source: Reprod Fertil. 2026 Aug 5;7(3):RAF250189. doi: 10.1530/RAF-25-0189 (PMC13452043; doi:10.1530/RAF-25-0189)
Supplement: Supplementary file 2 [file RAF-25-0189_supplementary_figure_2.pdf]

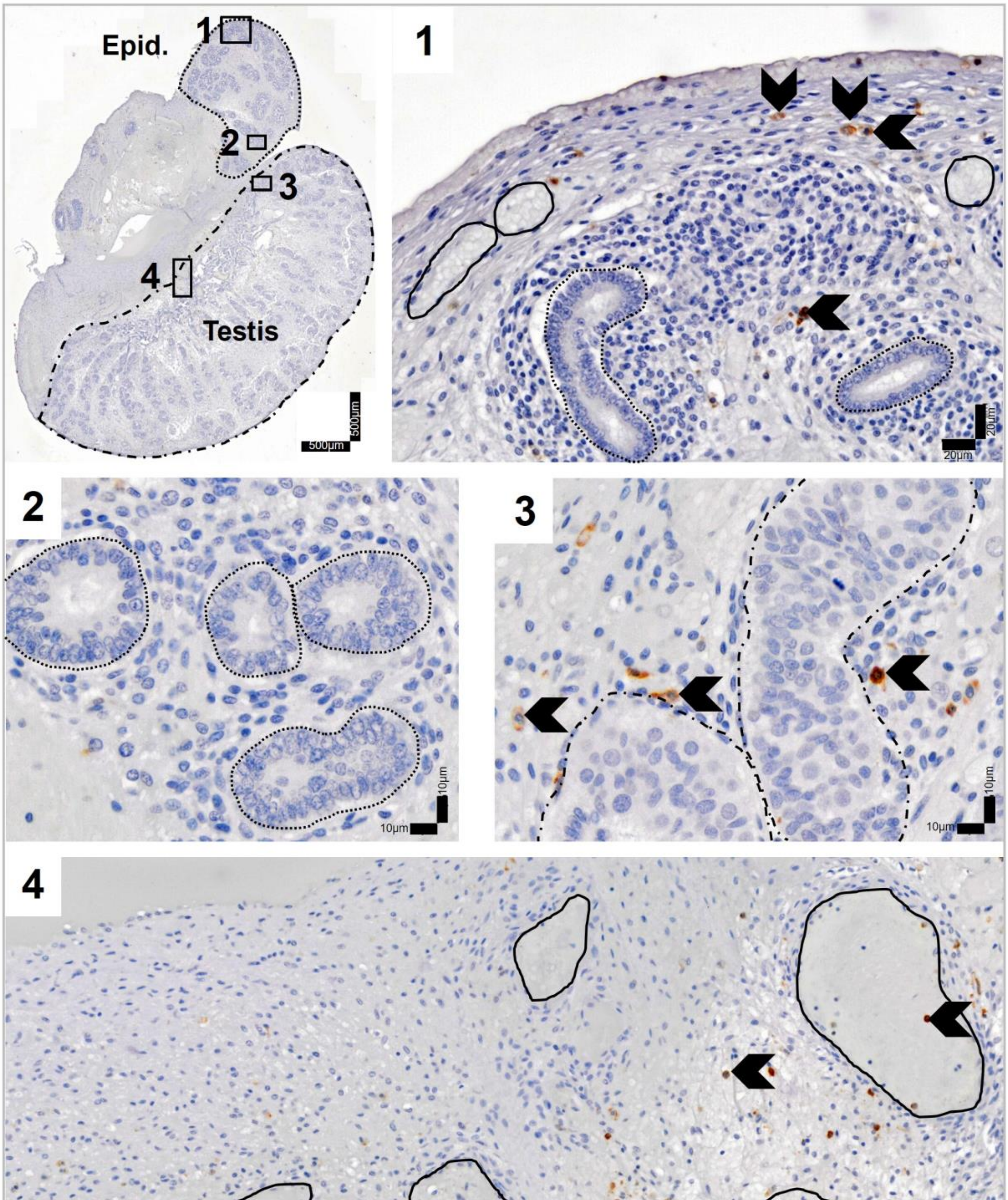

Epid.
1
2
3
4
Testis
500µm
500µm
1
20µm
20µm
2
10µm
10µm
3
10µm
10µm
4
10µm
10µm
