# Supplementary material for: Localisation of immune cell populations during human fetal testicular development
Source: Reprod Fertil. 2026 Aug 5;7(3):RAF250189. doi: 10.1530/RAF-25-0189 (PMC13452043; doi:10.1530/RAF-25-0189)
Supplement: Supplementary file 3 [file RAF-25-0189_supplementary_figure_3.pdf]

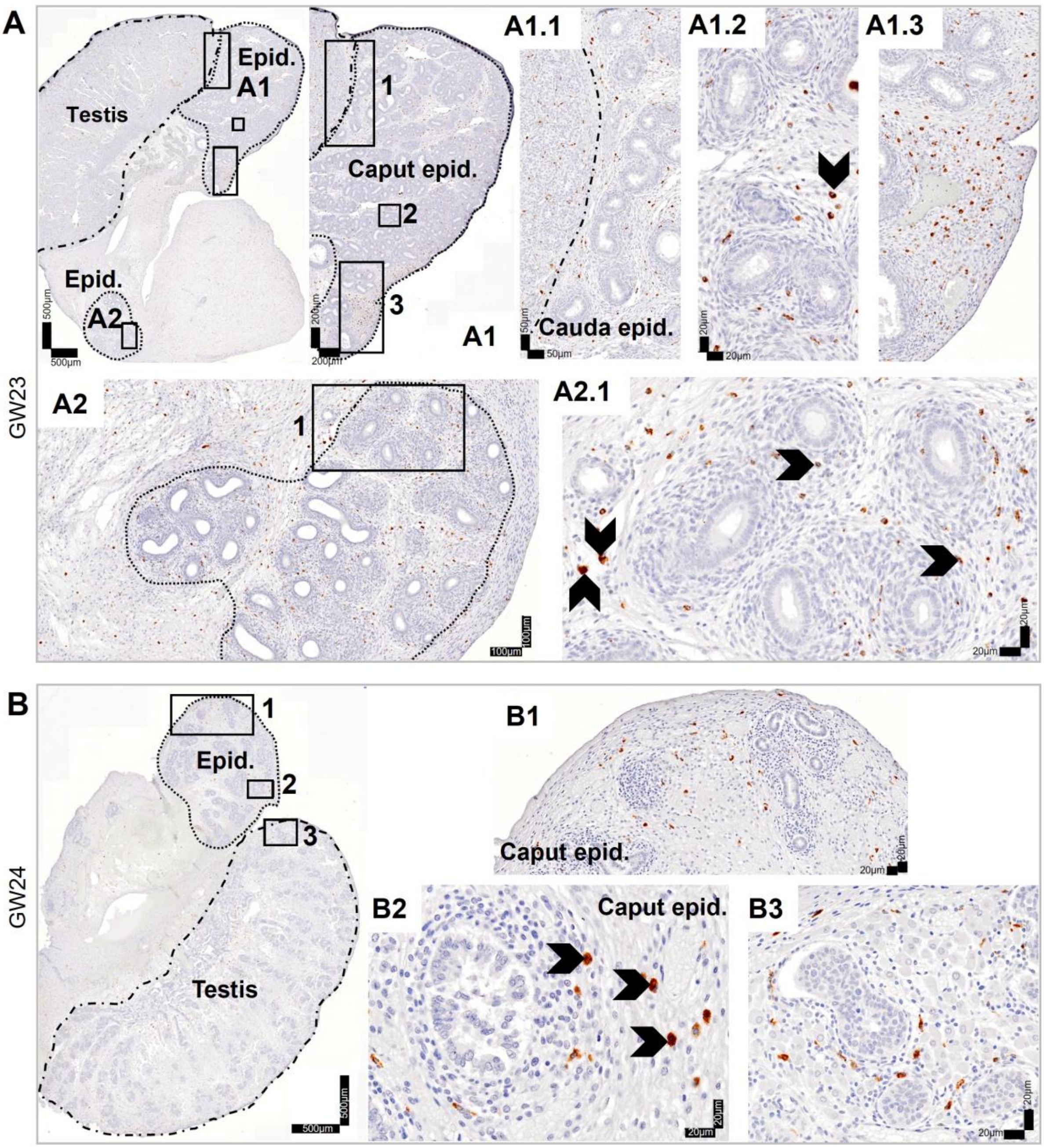
A
Testis
Epid.
A1
Epid.
A2
500µm
500µm
1
Caput epid.
2
3
200µm
200µm
A1.1
A1.2
A1.3
A1
Cauda epid.
50µm
50µm
20µm
20µm
GW23
A2
1
100µm
100µm
A2.1
20µm
20µm
B
1
Epid.
2
3
Testis
500µm
500µm
GW24
B1
Caput epid.
20µm
20µm
B2
Caput epid.
20µm
20µm
B3
20µm
20µm
