# Supplementary material for: Localisation of immune cell populations during human fetal testicular development
Source: Reprod Fertil. 2026 Aug 5;7(3):RAF250189. doi: 10.1530/RAF-25-0189 (PMC13452043; doi:10.1530/RAF-25-0189)
Supplement: Supplementary file 4 [file RAF-25-0189_supplementary_figure_4.pdf]

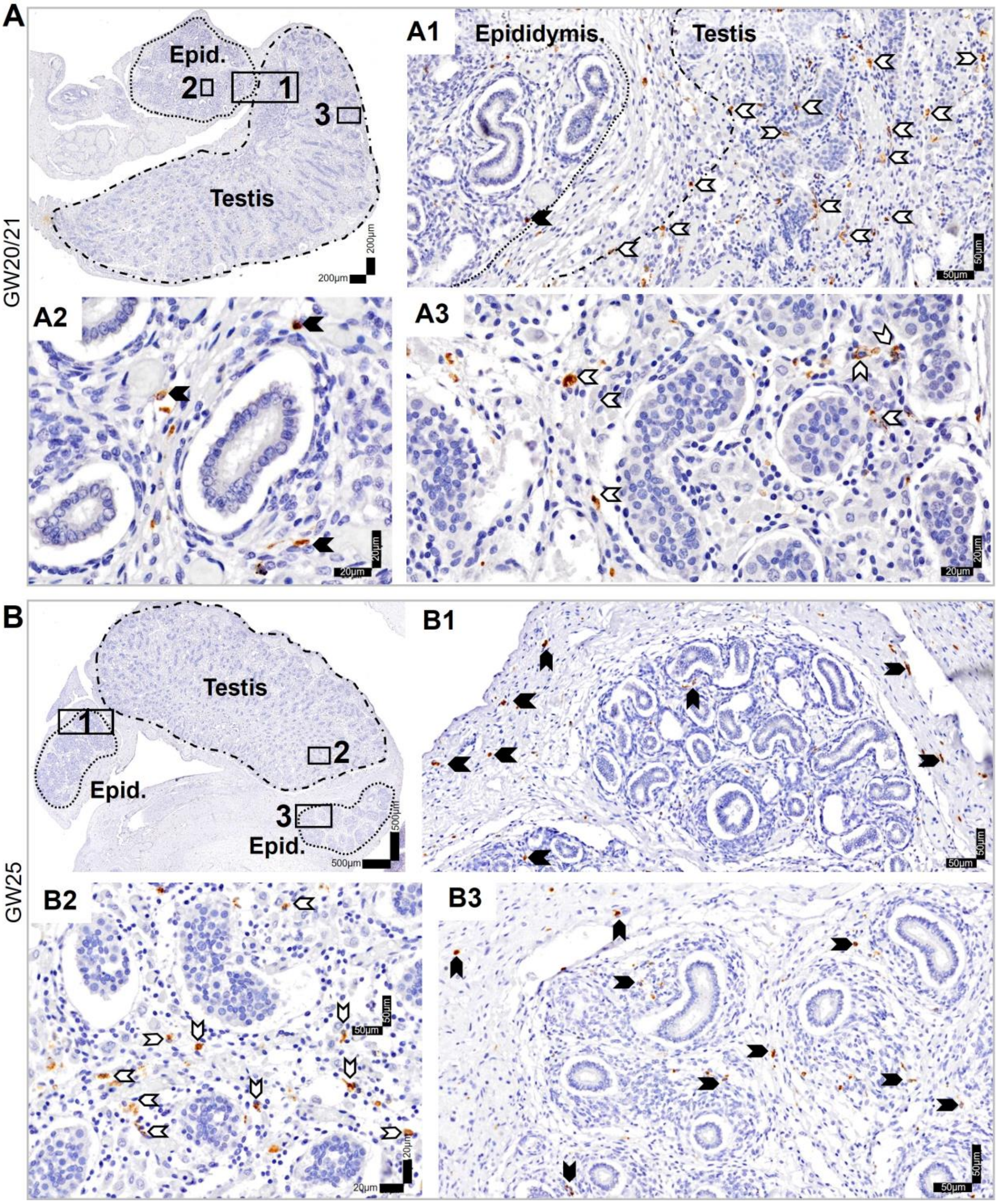
A
Epid.
2
1
3
Testis
200µm
200µm
GW20/21
A1
Epididymis
Testis
50µm
50µm
A2
20µm
20µm
A3
20µm
20µm
B
Testis
1
2
Epid.
3
Epid.
500µm
500µm
GW25
B1
50µm
50µm
B2
50µm
50µm
20µm
20µm
B3
50µm
50µm
