# Supplementary material for: Localisation of immune cell populations during human fetal testicular development
Source: Reprod Fertil. 2026 Aug 5;7(3):RAF250189. doi: 10.1530/RAF-25-0189 (PMC13452043; doi:10.1530/RAF-25-0189)
Supplement: Supplementary file 5 [file RAF-25-0189_supplementary_figure_5.pdf]

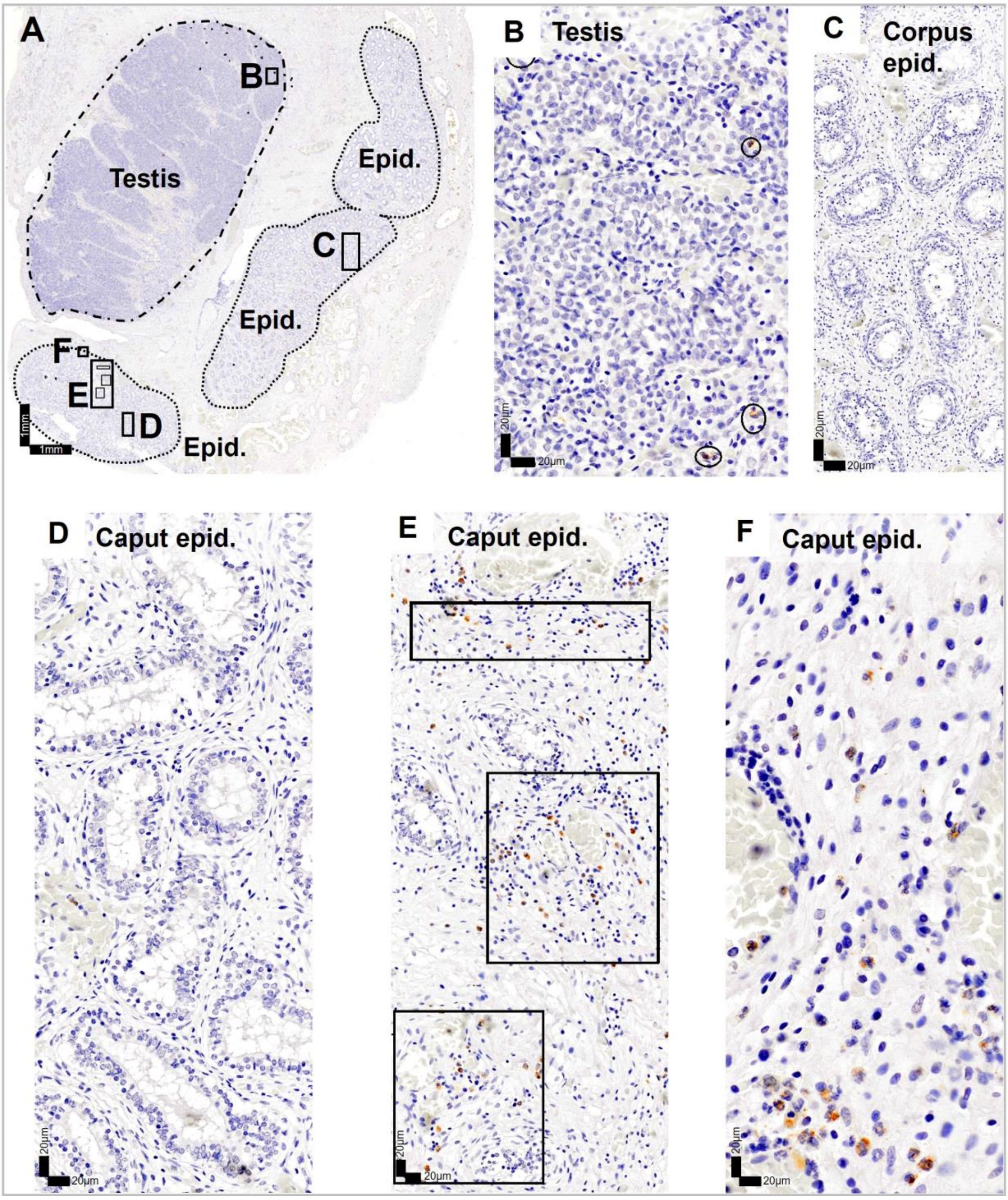
A
Testis
B
Epid.
C
Epid.
F
E
D
Epid.
1mm
1mm
B Testis
20µm
20µm
C Corpus epid.
20µm
20µm
D Caput epid.
20µm
20µm
E Caput epid.
20µm
20µm
F Caput epid.
20µm
20µm
