# Supplementary material for: Localisation of immune cell populations during human fetal testicular development
Source: Reprod Fertil. 2026 Aug 5;7(3):RAF250189. doi: 10.1530/RAF-25-0189 (PMC13452043; doi:10.1530/RAF-25-0189)
Supplement: Supplementary file 7 [file RAF-25-0189_supplementary_figure_7.pdf]

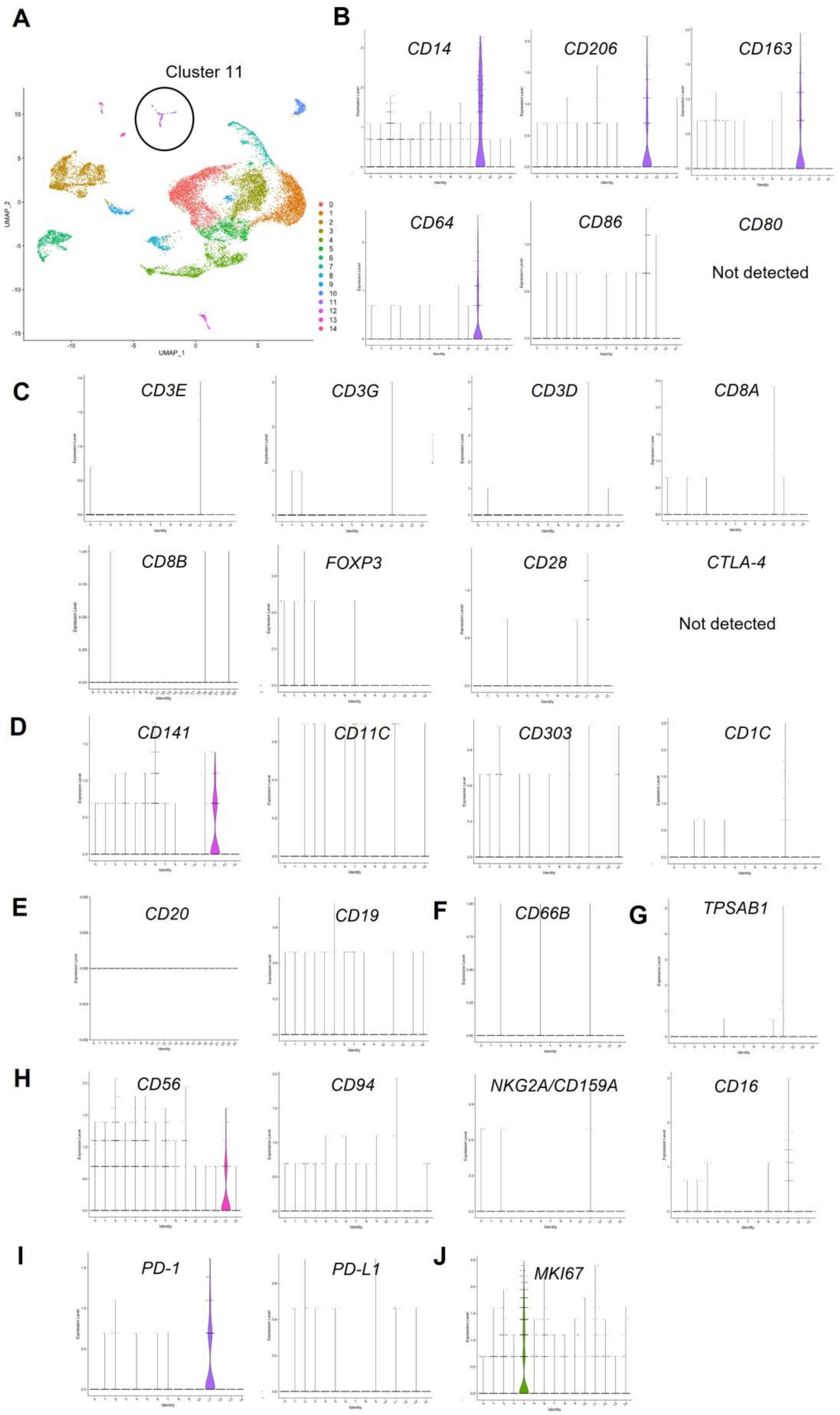

A
Cluster 11
UMAP_1
UMAP_2
B
CD14
CD206
CD163
CD64
CD86
CD80
Not detected
C
CD3E
CD3G
CD3D
CD8A
CD8B
FOXP3
CD28
CTLA-4
Not detected
D
CD141
CD11C
CD303
CD1C
E
CD20
CD19
F
CD66B
G
TPSAB1
H
CD56
CD94
NKG2A/CD159A
CD16
I
PD-1
PD-L1
J
MKI67
